# Case Report: Discovery a Novel SARS-CoV-2 Variant in a Six-Months Long-Term Swab Positive Female Suffering From Non-Hodgkin Lymphoma

**DOI:** 10.3389/fonc.2021.705948

**Published:** 2021-07-20

**Authors:** Ettore Capoluongo, Carmela Nardelli, Maria Valeria Esposito, Antonio Riccardo Buonomo, Monica Gelzo, Biagio Pinchera, Emanuela Zappulo, Giulio Viceconte, Giuseppe Portella, Mario Setaro, Ivan Gentile, Giuseppe Castaldo

**Affiliations:** ^1^ Department of Molecular Medicine and Medical Biotechnologies, University of Naples Federico II, Napoli, Italy; ^2^ CEINGE Biotecnologie Avanzate S.C.a R.L., Napoli, Italy; ^3^ Task Force on Microbiome Studies, University of Naples Federico II, Napoli, Italy; ^4^ Department of Clinical Medicine and Surgery, University of Naples Federico II, Napoli, Italy; ^5^ Department of Translational Medical Sciences, University of Naples Federico II, Napoli, Italy

**Keywords:** SARS-CoV-2, novel variant, whole genome sequencing, ORF3a, non-Hodgkin lymphoma, long-term infection

## Abstract

**Background:**

We report the case of a woman with non-Hodgkin lymphoma who remained positive on the molecular assay for SARS-CoV-2 for six months: she has never experienced a severe form of COVID-19 although in absence of seroconversion.

**Methods:**

The whole SARS-CoV-2 genome analysis was performed by the CleanPlex SARS-CoV-2 Research and Surveillance NGS Panel (PARAGON GENOMICS, Hayward, USA).

**Results:**

We found twenty-two mutations in SARS-CoV-2 genome and a novel deleterious ORF3a frameshift c.766_769del corresponding to a unique and novel lineage. The region affected by this frameshift variant is reported as being important in determining SARS-CoV-2 immunogenicity. Patient’s immunophenotype showed the absence of B lymphocytes and significantly reduced T-cell count. Only after the treatment with hyperimmune plasma she finally became negative on the swab.

**Conclusions:**

Our findings could be helpful in the management of patients with immunodeficiency, particularly when novel variants, potentially altering the virus immune response, are present.

## Introduction

Severe acute respiratory syndrome coronavirus 2 (SARS-CoV-2) is the causative agent of the current coronavirus disease 2019 (COVID-19) pandemic (1) with over 130 million people infected worldwide today (1, 2).

The SARS-CoV-2 virus contains a single positive stranded ribonucleic acid (RNA) of 30 kilobases, it contains 6 open reading frames (ORF1ab, ORF3a, ORF6, ORF7ab, ORF8, and ORF10) (3). Two-thirds of the virus genome comprises 1a/1b ORF and the remaining one-third of the genome code is used for M (membrane), S (spike), N (nucleocapsid), and E (enveloped) viral structural proteins (3).

In the past few months, several mutations have been associated to an altered SARS-CoV-2 infectivity, virulence, or host immune susceptibility (4, 5). These variants may affect the ability of antibodies generated through natural infection or vaccination to neutralize the virus. Some spike protein variants, such as the Asn501Tyr, His69_Val70del, and Glu484Lys ones, have independently emerged in many global strains, particularly those from the UK, South Africa, and Brazil: the latters are responsible of the pandemic resurgence when it appeared to be coming under control (6).

Here we report a case of a female patient with severe immunodeficiency who remained positive for six months on the molecular assay for SARS-CoV-2, since the first clinical and molecular diagnosis of COVID-19. Our findings show that she was carrying a unique viral strain corresponding to a completely new lineage. We believe that these findings could be helpful in evaluation of clinical feature of COVID-19 and viral characteristics.

The 64 years-old female, born in Campania region, is affected by arterial hypertension, potential occult HBV infection under prophylaxis with lamivudine and follicular variant of non-Hodgkin lymphoma, diagnosed in January 2018 and treated with cyclophosphamide, doxorubicin, vincristine, and prednisone (R-CHOP) chemotherapy. She was under maintenance therapy with subcutaneous rituximab 14 mg every two months, with last dose administered in June 2020.

She started complaining low-grade fever and dysgeusia on the 31st of August 2020. A nasopharyngeal swab (NFS) resulted positive for SARS-CoV-2 RNA on the 5th of September 2020. She was admitted to a peripheral hospital of the metropolitan area of Naples on the 26th of September for development of dyspnea and low peripheral oxygen saturation. At the admission, the high-resolution chest CT-scan (HRCT) showed bilateral ground-glass opacities (GGO) and peripheral bilateral consolidation. She was treated with intravenous methylprednisolone 20 mg BID, ceftriaxone 2 g i.v. QD and enoxaparin 4000 IU BID. A second HRCT evaluation was performed on the 14th of October, resulting in a significant reduction of the previously descripted parenchymal lesions. Nonetheless, at the beginning of December 2020, she started complaining low-grade fever and she showed a three-fold increase of C-reactive protein (CRP) level. Therefore, the third HRCT achieved, showed again bilateral increase of GGO. Since NFS continued to be positive for SARS-CoV-2 RNA, the patient was then transferred to Federico II University Hospital within the Infectious disease Department on date 11th of December 2020, for both the infectious disease and hematologic assessment.

On admission she was in good clinical conditions, good hemodynamic values, oxygen saturation of 93% on room-air, with PaO2 of 68 mmHg, low grade fever (Tmax 37.8°C). Omeprazole 20 mg QD, atorvastatin 10 mg QD, folic acid 14 mg QD, lamivudine 100 mg QD, olmesartan/hydrochlorothiazide 20/12.5 mg were administered.

Blood tests at the admission showed high glucose level (150 mg/dL), blood ferritin (860 ng/mL), LDH (319 U/L), PCR (1.43 mg/dL), WBC (4.230/mm3), with absolute lymphocyte count of 423/mm3, CD4+: 28% (absolute count: 118/mm3), CD8+: 50% (absolute count: 211/mm3) and CD56+ 10% (absolute count: 42/mm3); hemoglobin (10 g/dL) and normal neutrophil count.

Blood, urine, and sputum cultures, as well as serological test for intracellular bacteria, resulted negative for Legionella pneumophila and molecular assay for respiratory viruses. Due to the concomitant IgG hypogammaglobulinemia (IgG level 2.6 g/L), she received 20 g of intravenous immunoglobulins for two consecutive days.

The superficial and deep ultrasound on lymph nodes resulted normal: moreover, the hematologic consultation confirmed the complete remission from the disease and withheld further administration of rituximab until the resolution of COVID-19.

The patient was therefore scored as class four following the WHO clinical progression scale (7).

She was treated from the 11th to 17th of December with ceftaroline 600 mg BID and levofloxacine 750 mg QD, in a suspicion of bacterial superinfection, with no improvement. Two consecutive Beta-D-Glucan assays resulted negative.

On the 26th of December, another HRCT was performed, that showed bilateral GGOs with a severity score of 11/20 (8). A bronchoalveolar lavage fluid (BALF) was then collected two days later: galactomannan antigen, CMV-DNA, VZV-DNA, PCR for respiratory viruses, microscopic and bacteria, fungi and mycobacteria cultures were performed. GeneXpert assay for Mycobacterium tuberculosis resulted negative, while *P. jiroveci* direct immunofluorescence was positive. SARS-CoV-2 RNA was detected on BALF with a cycle threshold of 24. Trimethoprim-sulfamethoxazole (CTX) 20 mg/kg p.o. daily together with prednisone 20 mg BID were started, with improvement of blood gas exchanges and CRP reduction. Therapy with CTX was stopped after 21 days, with prednisone tapering.

A HRCT performed on the 26th of January 2021 showed improvement of GGOs, with a score of 6/20. Nonetheless, despite the clinical and radiologic improvement following the therapy for Pneumocystis pneumonia, our patient started to have low-grade fever, increased CRP, leukopenia and persistence of hypogammaglobulinemia, with IgG level of 4,4 g/dL. Thus, she received a further i.v. immunoglobulin administration 30 days apart from the previous. CMV DNA, beta-D-glucan, galactomannan resulted as negative on new repeated samples, with blood, sputum and urine cultures negative. On date 5th of February 2021, she was still recovered in the isolation hospital unit, in discrete respiratory conditions, still febrile, with increased CRP (last determination 2.3 mg/dL), negative procalcitonin, leukopenia (WBC 1790/mm3, Neu 870/mm3, lymphocytes 570/mm3) with confirmed positivity for SARS-CoV-2 RNA on NFS after 150 days from the first detection. The WBC cytometry typing on the peripheral blood, one week after the admission to our Medical School resulted in: absolute lymphocyte count (1043/mm3), with the absence of B-cells (i.e., 0/mm3), CD4+: 27% (absolute count: 282/mm3); CD8+: 58% (absolute count: 605/mm3) and CD56+: 7% (absolute count: 73/mm3).

She never developed anti-nuclear and anti-spike SARS-CoV-2 antibodies. The hematologic consultation for bone marrow examination was requested, together with SARS-CoV-2 RNA sequencing, to evaluate the presence of potential viral mutations associated to the persistence of detectable viral load. She could not yet be treated with rituximab, according to a board of hematologist from our hospital. On date 26th of February she was administered with hyperimmune serum-based therapy: two weeks later she finally became negative at NFS molecular assay.

## Material and Methods

All tests were performed within Lab n. 777777 of CEINGE Biotecnologie Avanzate S.C.a R.L., belonging to the CORONET Campania Regional network. Briefly, Real-time reverse transcriptase-polymerase chain reaction (RT-PCR) was performed by Allplex SARS-CoV-2 assay (Seegene, Seoul, Korea) as already reported by our group (9), according to manufacturer’s instructions, on the NSF deriving RNA of our patient. The study was approved by the Ethical Committee of the University Federico II of Naples (authorization n.180/20/ES1 on 25.05.2020) and was performed in compliance with the Declaration of Helsinki. The patient signed the informed consent to participate in the study.

Nasopharyngeal swab was collected using sterile cotton swab containing about 1 mL universal transport media (UTM - Copán, Brescia, Italy) and sent to our Lab in boxes at a controlled temperature within four hours from the samples collection.

Total RNA was extracted from 200 µL of NFS sample using a fully-automated system based on a Magpurix Viral/Pathogen Nucleic Acids kit (Zinexts, commercialized by Resnova, Genzano di Roma, Italy) running on a MAGPURIX 24 instrument, according to the manufacturer’s procedures. RNA was eluted in 50 µL elution buffer provided by the manufacturers. RNA quantity was evaluated through the NanoDrop 2000c spectrophotometer (Thermo Fisher Scientific, Waltham, MA, USA) and by using Qubit RNA HS assays kits (Life Technologies, Carlsbad, CA, USA).

Positive patient’s sample collected on 15 of February 2021 was used for the sequencing. Library preparation for sequence the entire SARS-CoV-2 genome was performed by the CleanPlex SARS-CoV-2 Research and Surveillance NGS Panel (PARAGON GENOMICS, Hayward, USA), according to the manufacturer’s instructions. The CleanPlex ^®^ SARS CoV 2 Research and Surveillance Panel was expertly designed from reference sequence MN908947 (NC 045512.2) using a proprietary design pipeline to cover the entire genome. Starting from purified RNA, the protocol allowed to generate target enriched NGS libraries using a three steps workflow with minimal tube to tube transfers by using multiplex PCR technology. The panel is two-pool designed to cover the entire SARS-CoV-2 genome. Briefly, the protocol provides the following steps: 1) cDNA synthesis and purification from purified RNA samples; 2) multiplex PCR reactions by target-specific primers to amplify the entire SARS-CoV-2 genome with the 2-pool design; 3) digestion reaction that achieves background cleaning by removing nonspecific PCR products; 4) PCR reaction by using CleanPlex Indexed PCR Primers. The library purification was performed using the AM-Pure XP beads (Beckman Coulter). The quality of prepared libraries was assessed by the TapeStation system (Agilent Technologies, Santa Clara, CA, USA) using the d1000 HS screen tape; then, library quantification was performed by using Qubit dsDNA HS assays kits (Life Technologies, Carlsbad, CA, USA).

The average peak observed for each library was 270 bp. Finally, libraries were pooled together for multiplexed sequencing. Libraries pool was diluted at a concentration of 10 pM and added to 10 pM PhiX control spike-in of 5% for low-diversity libraries, Sequencing reactions were carried out on the MiSeq instrument (Illumina, San Diego, CA, USA) using a PE 150 X 2 Micro flow cell to obtain an average coverage of about 1000X. Test was performed in duplicate.

The bioinformatics platform SOPHiA DDM v.4 (SOPHiA Genetics, Lausanne, Switzer-land) matching the samples against the reference sequence NC_045512.2 of SARS-CoV-2, was used to perform alignments, variant calling and quality filtering. Furthermore, Nextclade (https://clades.nextstrain.org/) and PANGO (https://cov-lineages.org/) free tools were used to identify the clade or lineage, respectively, which are defined by specific mutation signatures.

Immunophenotyping analysis was performed by multicolor flow cytometry as previously described by our group (10).

Hyperimmune plasma was provided by blood transfusion service of Federico II University Hospital and was obtained from healthy donors recovered from COVID-19 after standard preparation. Donors were selected based on a titer of neutralizing antibodies (determined though microneutralization assay) ≥1:160. The patient received three units of 300 mL of such plasma from 0-positive CcDee donor.

## Results

The genomic analysis of our COVID-19-ve patient showed the presence of several mutations in SARS-CoV-2 genome (**Table 1**).

**Table 1:** List of the 22 mutations identified in the patient’s SARS-CoV-2.

Ref Genome	Genome position	Gene	c.DNA	Coding Consequence	Protein	Protein
NC_045512.2	241	5’UTR	c.1-25C>T	5’UTR		
NC_045512.2	445	ORF1ab	c.180T>C	synonymous	p.(Val60=)	V60=
NC_045512.2	1513	ORF1ab	c.1248C>T	synonymous	p.(Cys416=)	C416=
NC_045512.2	2062	ORF1ab	c.1797C>T	synonymous	p.(Ala599=)	A599=
NC_045512.2	3037	ORF1ab	c.2772C>T	synonymous	p.(Phe924=)	F924=
NC_045512.2	4878	ORF1ab	c.4613C>A	Missense	p.(Thr1538Asn)	T1538N
NC_045512.2	5654	ORF1ab	c.5389C>T	synonymous	p.(Leu1797=)	L1797=
NC_045512.2	6286	ORF1ab	c.6021C>T	synonymous	p.(Thr2007=)	T2007=
NC_045512.2	14408	ORF1ab	c.14144C>T	Missense	p.(Pro4715Leu)	P4715L
NC_045512.2	19117	ORF1ab	c.18853G>T	Missense	p.(Ala6285Ser)	A6285S
NC_045512.2	21255	ORF1ab	c.20991G>C	synonymous	p.(Ala6997=)	A6997=
NC_045512.2	22227	S*	c.665C>T	Missense	p.(Ala222Val)	A222V
NC_045512.2	22346	S	c.784G>T	Missense	p.(Ala262Ser)	A262S
NC_045512.2	22377	S	c.815C>T	Missense	p.(Pro272Leu)	P272L
NC_045512.2	23403	S	c.1841A>G	Missense	p.(Asp614Gly)	D614G
NC_045512.2	25906	ORF3a	c.514G>T	Missense	p.(Gly172Cys)	G172C
**NC_045512.2**	**26157**	**ORF3a^§^**	**c.766_769del**	**Frameshift**	p.(Val256Ilefs*3)	**-**
NC_045512.2	26801	M	c.279C>G	synonymous	p.(Leu93=)	L93=
NC_045512.2	28560	N	c.287G>T	Missense	p.(Gly96Val)	G96V
NC_045512.2	28932	N*	c.659C>T	Missense	p.(Ala220Val)	A220V
NC_045512.2	29085	N	c.812C>T	Missense	p.(Thr271Ile)	T271I
NC_045512.2	29645	ORF10*	c.88G>T	Missense	p.(Val30Leu)	V30L

*The present three mutations are associated to the Italian lineage B.1.177.83 (11). ^§^c.766_769del (in bold) corresponds to a new variant never reported in the other databases (depth of coverage: 1700x).

**Figure 1:**
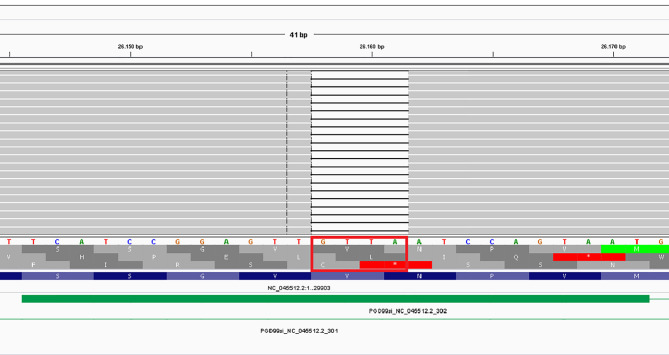
IGV representation of the c.766_769del affecting the ORF3a gene.

According to the PANGO lineages tool the analyses of NGS data enables us to identify the SARS-CoV-2 lineages with an assignment probability of 1 (100%): the lineage was unique and defined as B.1.177.83 defined as Italian lineage. The sequence of patient’s RNA showed 22 variants, of which three corresponded to those of the Italian lineage: the c.665C>T (S), c.659C>T (N) and the c.88G>T (ORF10) (11). Surprisingly, the novel ORF3a frameshift c.766_769del RNA variant identified a new clade at the bioinformatic analyses. This is the unique frameshift variant, determined by a 4 nucleotide (GTTA) deletion exiting in a premature stop codon (Val256Ilefs*3) of ORF3a domain. Details of the alignment of this deletion are reported in **Figure 1**, where the IGV tool shows the region involved and the consequence of the sequence alteration.

**Figure 2:**
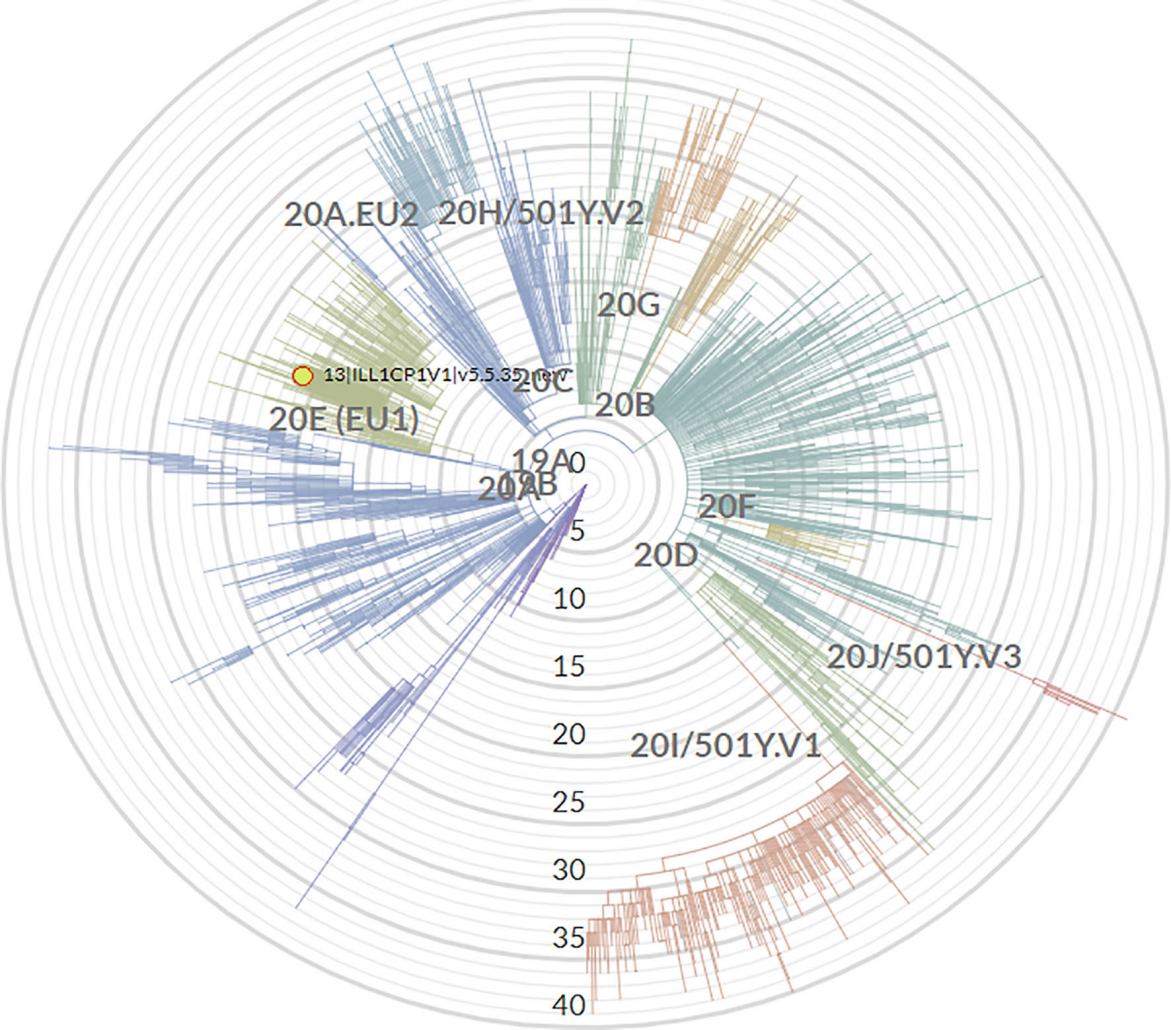
Phylogeny of SARS-CoV-2 clade obtained in our female patient.

In fact, both PANGO and Nextclade software indicated that this virus derives from clade 20E (EU1), but it differs from the initial 19A strain by 22 mutations. The phylogeny of our sequenced virus is reported in **Figure 2**. In order to verify if this clade was referred to the time of NFS withdrawal, we tried recovered the previous NFS tubes from the biobank of our hospital. We were able to obtain the samples collected on dates: 22 and 24 December 2020 plus that of 9th of February. By our NGS pipeline we obtained the same results, underlying that the patient mainly carried this unique clade.

Finally, as indicated by the red circle and the arrow, the clade differs by 22 mutations from that of Wuhan (19A) and by 11 mutations by the clade 20E (EU1).

## Discussion

To our knowledge, this is the first report describing a case of non-Hodgkin lymphoma patient who also resulted long-term positive for SARS-CoV-2. This finding is peculiar for the following reasons:

- The patient was immune-deficient (absence of B cells) due to her oncological disease: nevertheless, she did not suffer from severe symptoms COVID-19, although in presence or radiological features of bilateral ground-glass opacities (GGO) and peripheral bilateral consolidation; this could be also due to the very low number of Th activate cells (data not shown) and thus to the low production of cytokines;
- For about six months, a sort of balance between our variant (with a probably reduced immunogenic capacity) and the altered immunity of host was reached, determining the prolonged positivity at molecular NSF assay, which disappeared after the hyperimmune serum-based therapy was administered;
- NFS analyzed during the entire period of recovery resulted always positive for viral RNA, whilst the serum antibodies (IgM and IgG) titers continued to be undetectable (as expected, due the absence of B cells and the reduced T cell immunity). Nevertheless, it likely that this T-cell immunity was able to keep the virus under control in synergy with the various drug treatments administered to the patient.
- The main finding of the present case-report is the discovery of a novel variant, namely *ORF3a* c.766_769del frameshift. The latter is not reported in both the GISAID and other open-access databases. The peculiarity of this variant is that it falls within the ORF3a domain, a crucial region necessary for the best immune recognition by host immune system (12). The ORF3a corresponds to a group of accessory proteins (ORF3a, ORF6, ORF7a, ORF7b, ORF8 and ORF1) and consists of 275 AAs with its complete CDS appearing in 5527 isolates (13, 14). Among these, 2321 sequences have either no mutation nor only synonymous mutations, while 3206 ones carry insertion, deletion or nonsynonymous mutations (14). The authors reported that within MT358717 (USA: WA on 2020-03-27) and MT474130 (USA: CA on 2020-03-31), at positions V256 and N257, respectively, a point deletion occurred (14). The prediction associated to both missense and other non-synonymous alteration within this particular ORF3a region indicate significant deleterious effects (15). So, we can extrapolate that if only one more two amino acid changes determine deleterious effect, a frameshift deletion in the same region which produce the truncation of the remaining protein, can result in the same or more damaging consequence. Further data obtained on more than 70,000 SARS-CoV-2 sequenced genomes revealed that most of the ORF3a variants correspond to a single point mutation, while four variants are distinguished by co-occurrence of two mutations, one of which is always Q→H in position 57 of the ORF3 reference sequence (16). The frequency of Q57H variant results constantly high in overall cohorts of patients analyzed, while that of other mutations varies. Moreover, ten out of the seventeen mutant sites occur within the ORF3a transmembrane domain, where four of these contain the mutation Q57H paired with other amino acidic changes (A99V, S58N, Y264C, G172V) (16). Interestingly, only in two cases the associated mutations fall within the extracellular domain (G172V and Y264C). Among these mutations, the variant G251V is consistently highly frequent and has been isolated all over the world: very surprisingly, all these variants are absent in our sequenced viral genome. We underline as close to our p.(Val256Ilefs*3) mutation, the G254R was found to result in decreased intensity of specific epitopes (17). In addition, literature reported as within the ORF3a epitope, corresponding to peptide 4 (249–257), only three mutant residues are present (17). Our deletion is the first reported in this domain and we can speculate that the final effect can be similar to that of the G254R. We can hypothesize that combination of the following factors: a) the reduced capability of patient’s response associated to the primary immune suppression given by the lymphoma, and b) the decreased intensity of specific epitopes exposition, resulted in the absence of immune-response in a non-severely symptomatic COVID-19 patient.

We underline that cell surface localization of ORF3a in SARS-CoV strengthens viral entry within the host and has immunogenic properties (12). ORF3a is also implicated in ion channel formation, modulating the release of virus from the host cell (13). Interestingly, SARS-CoV-2 ORF3a mutations are reported as associated with higher infection and mortality rate (12). Nevertheless, our patient showed a significant reduction of both T and depletion of B cells, with possible effects on mechanisms leading to cytokine storm, the latter being one of the causes promoting severe symptoms in COVID-19 patients. We underline that Majumdar et al. (12) reported results based on aggregate data rather than on single patients: they concluded, indeed, that changes in amino acid residues are likely to interfere with the biological role of WT SARS-CoV-2 ORF3a protein and need to be evaluated in context of host−pathogen interaction. Therefore, we can only argue as, in peculiar cases, the clinical history of single patient can diverge from that of aggregate patients’ cohorts, often not homogenous, underlying as the investigation on virus characteristics should be deepened in the context of patient’s feature, better tailor her/his management. Furthermore, SARS-CoV-2 ORF3a protein is widely expressed in intracellular and plasma membranes, that trigger apoptosis and inflammatory within both the infected and transfected cells, respectively (18, 19). Interestingly, ORF3a presents a TRAF3-binding motif able to activate the NLRP3 inflammasome and it is a po-tent stimulator of pro-IL-1β gene transcription (20). In animal models of SARS-CoV infection, deletion of ORF3a gene reduced virus replication (21). Importantly, significant CD4+ and CD8+ T cell responses to SARS-CoV-2 in infected individuals are directed against ORF3a: therefore, the absence of three amino acids within a crucial immunogenic region of ORF3a can emphasize the potential capability of this variant to escape the immune system and spread within population. Noteworthy, the patient was able to eradicate the NFS virus positivity only after the infusion of hyperimmune plasma.

We can indeed highlight that the absence of circulation of this variant in our Campania Region, such also in other countries, can be the effect of the long period of hospitalization in isolation of our patient, which was also preceded by a long period of isolation at home. These two conditions did not allow the spread of this variant not only within the family, but also in the Italian population. Data obtained by using the GISAID, Nextclade and PANGO databases, confirm the unicity of our variant since the mutations mainly recurrent in ORF3a region are not present in our isolate.

The present paper lacks functional study of this deletion, that unfortunately drops within a region that is reported as being unmodeled (22).

Finally, we have completely sequenced fifty viral samples corresponding to individuals recovered in our academic hospital, but we did not find this variant in other subjects with other forms of COVID-19: therefore, we cannot verify if the absence of immunoglobulin seroconversion in our patient is related to the general immune suppression related to non-Hodgkin lymphoma rather than to the SARS-CoV-2 altered/decreased intensity of specific epitopes presentation. Nevertheless, it is reported that the immunocompromised individuals are more likely to produce variants due to long-term virus replication, which is probably due to the selective pressure from therapeutic such antibody cocktails (23). We cannot exclude that the prolonged positivity on NFS have been amplified by concomitant rituximab therapy (24, 25). Nevertheless, differently from findings reported by Esmaeili et al. (24), rituximab administration was not associated with higher risk of severe side effects and more severe WHO clinical progression scale. It is however reported a prolonged SARS-CoV-2 infection in patient und rituximab regimen (25). Among the limits of our paper, we can report the following: a) only a late timepoint was analyzed for this patient in terms of virus sequencing: therefore, we it cannot formally be proven that variation originated in this specific individual, although it is likely. In this regard, we underline as methods of massive parallel sequencing have not been immediately implemented in laboratory tracing in our region as it happened either in other Italian areas or countries (26, 27): b) the functional impact of our alteration has been extrapolated by data regarding other point alteration falling in the same domain. Further studies have been planned to better characterize this new variant, possibly in animal or cellular models: these experimental procedures take time and are also expensive.

This new variant has been deposited in GeneBank as the “*Corradino*” variant, in memory of this unfortunate member of the Hohenstaufen family, to which Frederick II, founder of our university, also belongs (GenBank: MZ054387). Finally, patient have benefited from the long period of recovery and the isolation within our hospital department. In this regard we underline as the patient’s isolation prevented her from spreading this “private” strain in people close to her (family members) and, consequently, in the population, with important repercussions on public health. Therefore, in cases similar to this one, the tracing of the virus isolated by NGS should be strongly suggested.

## Data Availability Statement

The datasets presented in this study can be found in online repositories. The names of the repository/repositories and accession number(s) can be found below: GenBank, MZ054387.1.

## Ethics Statement

The studies involving human participants were reviewed and approved by Ethical Committee of the University Federico II of Naples. The patients/participants provided their written informed consent to participate in this study.

## Author Contributions

EC: Conceptualization, writing-original draft, and writing-review and editing. CN, ME: Investigation, formal analysis, and writing-original draft. AB, BP, EZ, GV, GP, IG: Resources. MG, MS: Investigation. GC: Funding, writing-review and editing. All authors contributed to the article and approved the submitted version.

## Funding

This research was funded by a grant of Regione Campania, Task Force COVID-19 DGR 140/17 March 2020.

## Conflict of Interest

The authors declare that the research was conducted in the absence of any commercial or financial relationships that could be construed as a potential conflict of interest.
